# My sweetheart has a basketball match: The relationship between love-hate and identification of individuals attending a euroleague match for recreational purposes

**DOI:** 10.1371/journal.pone.0307892

**Published:** 2024-07-26

**Authors:** Mustafa Can Koc, Laurentiu-Gabriel Talaghir, Aydin Pekel, Arif Cetin, Leonard Stoica

**Affiliations:** 1 School of Physical Education and Sports, Istanbul Gelisim University, Istanbul, Türkiye; 2 Directorate of Sports Sciences Application and Research Center, Istanbul Gelisim University, Istanbul, Türkiye; 3 Faculty of Physical Education and Sport, Dunarea de Jos University, Galati, Romania; 4 Faculty of Sport Science, Marmara University, Istanbul, Türkiye; University of Barcelona: Universitat de Barcelona, SPAIN

## Abstract

The objective of this research was to examine the Love-Hate and Identification Relationship of Individuals Participating in Euroleague Match for Recreational Purposes. The study was conducted using a relational survey methodology. The study’s population comprises persons who watching recreational purpose part in the Euroleague match held in Istanbul in 2023–2024 season, while the sample consists of 178 voluntary participants selected through convenience sampling. The participants completed the Fan Love-Hate Scale and Fan Identification Scale, in addition to being asked about their gender, marital status, age, educational status, and frequency of attending football matches per week. The data collected from the personal information form and scales was entered into the IBM SPSS 24.0 software package for analysis. Statistical analyses were conducted using the Independent Sample T test and One-way Anova methods. The LSD test was employed to ascertain the dissimilarity between the groups. The Pearson correlation analysis was utilized to ascertain the association between the variables of love-hate and identity. In summary, it is evident that demographic factors, including gender and age, significantly influence fan perceptions and sports identification. In contrast, there is no substantial correlation observed between attributes such as level of education achieved and the frequency of engaging in sports activities, and the aforementioned outcomes. The significant associations identified between the Fan Love-Hate Scale and the Sports Fan Identification Scale underscore the complex relationship between fans’ emotional experiences and their connection to sports. Further investigations could be conducted to go deeper into the underlying causes that contribute to these relationships and inequalities, so resulting in a more thorough understanding of fan psychology.

## 1. Introduction

In present-day nations, the notion of sport is widely recognized as a significant component of the leisure industry and is increasingly exerting its influence on society [1]. This phenomenon is concurrent with the evolution and transformation of the notion of enthusiasts. Efficient time management is a crucial aspect of individuals’ daily life practices [2]. The primary focus of this period entails assessing the leisure time that falls outside of mandatory obligations. Different individuals may have different preferences when it comes to engaging in various activities. In this discussion, our primary focus will be on Euroleague basketball, as we explore other recreational hobbies. The Euroleague is a basketball organization renowned for its exceptional degree of spectator enjoyment, entertainment value, and fervor. The significance of this study lies in its focus on Euroleague participants as a kind of recreational activity in the realm of sports, as there is a dearth of existing research on this subject in the academic literature. Leisure time, a precious resource in individuals’ lives, encompasses the duration required within the fast-paced and demanding nature of everyday existence. The notion of leisure time, commonly situated within the temporal framework, is commonly understood as the duration remaining after engaging in job activities [3]. The term "Licere" serves as the etymological root for the English word "Leisure" and carries the connotation of "Permission" in the Latin language [4]. Upon careful examination of the existing literature, it becomes evident that the scholarly research pertaining to hatred in sports predominantly centers around the phenomenon of fan-based animosity [5–7]. The phenomenon of fan hatred, which is a prevalent emotional response, particularly within the context of spectator sports, has been the subject of research investigations, mostly focusing on spectator sports [8]. Fan animosity refers to the amalgamation of adverse attitudes, behaviors, and unpleasant feelings exhibited by an individual against the players, managers, and fan community of a rival team [9, 10]. When an individual develops aversion towards the constituents of an opposing team, they are inclined to exhibit acts of violence and hostility, which can be attributed to the resultant effects of animosity. Furthermore, when becoming a fan, individuals experience a shift in emotions, characterized by a stark contrast between the animosity directed towards the competitor team and the subsequent display of actions. This transformation is accompanied by the cultivation of affectionate sentiments towards the team being supported, exemplified by the emergence of loyalty and passion [11]. Love can be conceptualized as a set of behaviors that encompass responsibility, respect, understanding, and attachment [12]. Fan love encompasses a confluence of affective states that symbolize the intimate, genuine, elated, and yearning sentiments experienced by fans towards the sports team they endorse [5]. When an individual experiences affection towards their sports team, they tend to display actions associated with loyalty. In the academic literature, these behaviors can be characterized as follows: individuals engage in the act of observing their favored team’s matches either through televised broadcasts or by physically attending the matches at the stadium, irrespective of the matches’ significance. Furthermore, they demonstrate unwavering support for their team, not only during victorious moments but also in the face of undesirable outcomes such as defeats [13]. This support remains steadfast regardless of their team’s poor performance throughout a season, losses to their primary rivals, or the absence of star players [14, 15]. Additionally, individuals may experience feelings of anger and exhibit aggressive behaviors towards both the opposing team and its supporters. They may also exhibit their allegiance by purchasing products adorned with their team’s logo or other symbols associated with the team, subsequently displaying these items in their homes or workplaces [14, 16]. In contemporary times, the sport of basketball has witnessed a notable increase in both participation and spectatorship. The present scenario has brought the concepts of fan and identification into prominence [17]. According to TDK, the term "fan" refers to an individual who has a strong emotional attachment to the colors, club, or flag associated with a particular athlete or group of athletes. In contrast, a sports spectator refers to an individual who observes a sporting event or contest [18]. In essence, a sports spectator can be defined as an individual who observes a game or sports events by physically attending the venue where the event takes place [19]. The term "supporters" pertains to persons who endorse a specific group, individual, or set of views [20]. From a social identity perspective, individuals who become part of a group gain a sense of belonging and create more emotional connections [21]. The research route can be explained objectively and concisely. The literature has definitions pertaining to the notion of team identification [22]. define team identification as individuals being related to other team members and having a psychological bond with the team, while [23] define team identification as psychological and behavioral commitment. Furthermore, [18] provide a more precise definition of team identification, characterizing it as the extent to which spectators perceive the triumphs and failures of their preferred team as personal victories and defeats. Given the available data, the primary objective of this study is to examine the complex dynamics of the love-hate and identification phenomena among those who engage in recreational basketball game viewing.

## 2. Materials and methods

### 2.1. Research model

In this study, it was aimed to examine the Love-Hate and Identification Relationship of Individuals Participating in Euroleague Match for Recreational Purposes. The study was conducted using a relational survey methodology. The relational survey model can be described as a paradigm that seeks to ascertain the presence and/or extent of variation between multiple variables [24].

### 2.2. Population and sample

The population of this study consists of individuals who attended a Euroleague match in Istanbul during the 2023–2024 season for entertainment purposes. The event was chosen because of the high popularity of the Euroleague and its ability to attract diverse groups of spectators attending for entertainment purposes. This selection provides a broad representation of recreational sports spectators, which is crucial for the study’s aim of understanding the relationship between love-hate and identification among these individuals. The sample for this study was selected using convenience sampling method. Convenience sampling was chosen due to the practical considerations of accessing participants who were readily available and willing to participate during the match events. This method, while limiting the generalizability of the findings to the broader population, is appropriate for exploratory studies aimed at understanding complex social and psychological dynamics in specific contexts [25]. Given the exploratory nature of the study and the specific context of Euroleague match attendees, this method allowed for the efficient gathering of relevant data within a limited timeframe. Data collection was conducted on January 18, 2024, ensuring that the study captured a cross-section of match attendees’ immediate post-game reflections and emotions. On the day of the match, a team of trained researchers approached attendees at various points within the venue, informing them about the purpose of the study and inviting them to participate. Those who agreed to participate were provided with a questionnaire package including the Personal Information Form, Fan Love-Hate Scale, and Fan Identification Scale. A total of 198 questionnaires were distributed. After excluding incomplete responses, a final sample of 178 valid responses was obtained.

### 2.3. Research process

An application for this research was submitted to the Ethics Committee Unit of Istanbul Gelisim University on 16th October 2023. The Ethics Committee approved (Approval Code:2024–01) this study on 12st January 2024. Data was collected on 18th January 2024 from funs who attended the Anadolu Efes-Barcelona basketball match played in Istanbul.

According to the data presented in Table 1, the majority of participants, specifically 68.0%, were identified as male, while 32% were identified as female. Additionally, 53.4% of the participants reported being single, while 46.6% reported being married. The study revealed that 12.9% of the participants fell within the age range of 18–25, while 33.7% were aged between 26–33. Additionally, 27.5% of the individuals were found to be between the ages of 34–41, while the remaining 25.8% were aged 42 and above. The study revealed that 16.3% of the individuals surveyed had completed high school, 8.4% had obtained an associate degree, 56.2% had completed university, and 19.1% had achieved master’s degree. When respondents were queried about the frequency of their attendance at football matches each week, the majority, comprising 74.2% of the sample, indicated a preference for attending matches on a single day. A smaller proportion, constituting 18% of the respondents, reported attending matches on two days. Lastly, a minority, accounting for 7.9% of the participants, indicated a preference for attending matches on three or more days per week.

**Table 1:** Demographic characteristics of the participants.

Variables	Groups	N	%
**Gender**	Female	57	32,0
	Male	121	68,0
	**Total**	**178**	**100,0**
**Marital Status**	Single	95	53,4
	Married	83	46,6
	**Total**	**178**	**100,0**
**Age**	18–25 years	23	12,9
	26–33 years	60	33,7
	34–41 years	49	27,5
	42 years and above	46	25,8
	**Total**	**178**	**100,0**
**Educational Level**	High School	29	16,3
	Associate degree	15	8,4
	University	100	56,2
	Master’s degree	34	19,1
	**Total**	**178**	**100,0**
**What is the frequency of your attendance at match viewings on a weekly basis?**	1 day	132	74,2
	2 days	32	18,0
	3 days and above	14	7,9
	**Total**	**178**	**100,0**

### 2.4. Data collection tools

The study employed the use of many data collection tools, including the "Personal Information Form" which gathered information on gender, marital status, age, educational status, and frequency of attending football matches. Additionally, the "Fan Love-Hate Scale" and "Fan Identification Scale" were utilized to collect relevant data.

### 2.5. Fan love-hate scale

The ’Fan Love-Hate Scale’, first established by [5] and then modified into Turkish by [26], was employed in the study. The scale comprises seven items and is evaluated using a 10-point Likert scale ranging from "Strongly Disagree" to "Strongly Agree". The Hate Subdimension is measured on a scale ranging from 1 to 4, while the Love Subscale is also assessed on the same scale. The construct comprises two sub-dimensions, each containing pieces numbered 5, 6, and 7. The scale is additionally assessed and quantified as a cumulative score.

### 2.6. Fan identification scale

The researchers [27] developed the sport fan identification scale, which was then adapted into Turkish by [28]. The scale, comprising of seven items and one dimension, was utilized for the assessment of the participants. The scale is assessed on a range of 1 to 8 points. The obtaining of high scores on the scale suggests a high level of identification. The original scale demonstrates a high level of internal consistency, as indicated by a Cronbach Alpha coefficient of .91. Additionally, the average item-total correlation is 59.

### 2.7. Data analysis

The acquired data were inputted into the Spss 25.0 software package, and the subsequent analyses were conducted using this program. The candidates’ personal information, as well as the scale total scores and factor scores, were obtained by assessing the frequency levels (f) and percentages (%) values. The examination of the scores involved an analysis of the normal distribution through the use of normal distribution curves, skewness-kurtosis values, histograms displaying normal distribution curves, and the use of the Kolmogorov-Smirnov test for group sizes over 50. The study employed many statistical techniques, including distribution analysis, descriptive analysis, parametric hypothesis tests, independent sample T-test, and one-way Anova analysis. The post-hoc analysis employed to ascertain the distinction between groups involved the utilization of the Least Significant Difference (LSD) test. This particular technique was chosen due to its conservative nature, which surpasses the pairwise comparison tests commonly employed in the comparison of groups with homogeneous variances. The LSD test is particularly suitable for detecting small differences and can be applied to groups with homogeneous variances but varying sample sizes [29]. The study employed Pearson Correlation Analysis to ascertain the association between Love-Hate and Identification. During the statistical analysis and interpretation of the data, a significance level of 0.05 was utilized.

## 3. Results

Upon examination of the normal distribution curves of the scales in Table 2, it was ascertained that there were no departures from normality. Furthermore, while considering the skewness and kurtosis coefficients, it was seen that all scores fell within the range of ±1. According to **[**30**]**, a normal distribution can be inferred if the skewness and kurtosis coefficients of the variables fall within the range of ±1.5. In contrast, [19] contends that the presence of values falling within the range of ±1 indicates a lack of significant departures from normality. Given that the skewness and kurtosis values of the scores did not exhibit extreme levels and fell within the range of ±1, while also lacking severe deviations in the normal distribution curves, the decision was made to employ parametric statistical analysis tests.

**Table 2:** Normality analysis of the scales.

	Dimensions	N	Skewness	Kurtosis	p
**Fan Love-Hate Scale**	**Hate**	178	,611	-,952	,059
	**Love**	178	,088	,179	,115
	**Total Score**	178	,416	-,795	,049
**Fan Identification Scale**	**Total Score**	178	-,448	-,817	,089

Upon examination of Table 3, it becomes evident that there exists a statistically significant variance in the hate, love sub-dimensions, as well as the overall score, of the fan love-hate scale when considering gender as a variable.

**Table 3:** Evaluation of scales according to gender.

		Variables	N	X± Ss	t	p
**Fan Love-Hate Scale**	**Hate**	Female	57	10,23**±**7,86	-5,186	,000**
		Male	121	20,24**±**13,53		
	**Love**	Female	57	24,28**±**5,77	-4,410	,000**
		Male	121	27,66**±**4,73		
	**Total**	Female	57	33,60**±**9,72	5,806	,000**
		Male	121	46,92**±**15,97		
**Fan Identification Scale**	**Total**	Female	57	29,81**±**11,58	-6,950	,000**
		Male	121	42,93**±**12,11		

*p<,050

**p<,001

Upon examination of Table 4, it is evident that there exists no statistically significant disparity in the sub-dimensions of hate and love within the fan love-hate scale based on marital status. However, a discrepancy is observed in the overall score. The analysis revealed that there was no statistically significant variation in the degree of sports fan identification based on the variable of marital status.

**Table 4:** Evaluation of scales according to marital status.

		Variables	N	X±Ss	t	p
**Fan Love-Hate Scale**	**Hate**	Single	95	15,69**±**12,29	1,948	,138
		Married	83	18,57**±**13,40		
	**Love**	Single	95	25,94**±**5,94	4,937	,188
		Married	83	27,04**±**5,03		
	**Total**	Single	95	40,49**±**15,69	,016	,047*
		Married	83	45,1215,09		
**Fan Identification Scale**	**Total**	Single	95	36,29**±**13,98	2,207	,009*
		Married	83	41,51**±**12,19		

*p<,050

**p<,001

Upon analysis of Table 5, it becomes evident that there exists a discernible disparity in the hate sub-dimension of the Fan Love-Hate Scale based on the age demographics of the individuals. The observed discrepancy can be attributed to the distinction between the age groups of 26–33 years and 30–35 years, as well as the age group of 26–33 years and individuals aged 36 and above. The analysis revealed that there was not a statistically significant disparity in the love dimension. A disparity in the overall score of the sports fan identification scale was found based on the age variable. The observed discrepancy can be attributed to the distinction between those aged 26–33 and those aged 30–35, as well as the distinction between individuals aged 26–33 and those aged 36 and above.

**Table 5:** Evaluation of the scales according to the age of the participants.

		Yaş	N	X±Ss	F	p	Difference (LSD)
**Fan Love-Hate Scale**	**Hate**	18-25^a^	23	16,78±10,80	2,753	,044*	c>b
		26-33^b^	60	13,48 ±10,94			
							d>b
		30-35^c^	49	18,53 ±13,55			
		36 and above^d^	46	20,20± 14,56			
	**Love**	18-25^a^	23	27,43±4,57	,408	,748	-
		26-33^b^	60	26,15±5,34			
		30-35^c^	49	26,39 ±5,93			
		36 and above^d^	46	26,91± 5,00			
	**Total**	18-25^a^	23	42,83± 11,99	1,979	,119	-
		26-33^b^	60	38,88± 13,73			
		30-35^c^	49	44,37± 16,51			
		36 and above^d^	46	45,65 ±17,63			
**Fan Identification Scale**	**Total**	18-25^a^	23	37,35± 10,98	3,460	,018*	c>b
		26-33^b^	60	34,77±14,36			
							d>b

		30-35^c^	49	42,20± 11,25			
		36 and above^d^	46	40,87 ±14,20			

*p<,050

**p<,001

Upon examination of Table 6, it is evident that there exists no statistically significant disparity between the sub-dimensions of the fan love-hate scale and the overall score of the sports fan identification scale with respect to the educational background of the participants.

**Table 6:** Evaluation of the scales according to the education level of the participants.

		Education Level	N	X±Ss	F	p	Difference (LSD)
**Fan Love-Hate Scale**	**Hate**	High school^a^	29	20,52±15,27	,881	,452	-
		Associate degree ^b^	15	16,47±14,83			
		University^c^	100	16,13±11,80			
		Master’s degree ^d^	34	16,97±12,87			
	**Love**	High school^a^	29	27,86±4,47	,673	,570	-
		Associate degree ^b^	15	26,33±6,02			
		University^c^	100	26,35±5,33			
		Master’s degree ^d^	34	26,26±5,65			
	**Total**	High school^a^	29	48,21±16,35	1,517	,212	-
		Associate degree ^b^	15	42,47±17,85			
		University^c^	100	41,61±14,22			
		Master’s degree ^d^	34	41,06±17,16			
**Fan Identification Scale**	**Total**	High school^a^	29	43,17±11,41	1,290	,280	-
		Associate degree ^b^	15	37,73±14,52			
		University^c^	100	37,99±13,50			
		Master’s degree ^d^	34	37,53±13,98			

*p<,050

**p<,001

Upon examining Table 7, it is evident that there exists no statistically significant disparity between the sub-dimensions of the fan love-hate scale and the overall score of the sports fan identification scale, in relation to the variable denoting the frequency of attending sporting events on a weekly basis.

**Table 7:** Evaluation of the scales according to the variable of how many days a week do you go to watch sportive activities.

			N	X±Ss	F	p	Difference (LSD)
**Fan Love-Hate Scale**	**Hate**	1 day^a^	132	16,92**±**12,86	,193	,824	-
		2 days^b^	32	18,09**±**13,09			
		3 days and above^c^	14	15,64**±**13,21			
	**Love**	1 day^a^	132	26,35**±**5,49	,106	,900	-
		2 days^b^	32	26,63**±**5,83			
		3 days and above^c^	14	27,00**±**5,74			
	**Total**	1 day^a^	132	42,19**±**15,91	,343	,710	-
		2 days^b^	32	44,72**±**14,28			
		3 days and above^c^	14	42,29**±**15,47			
**Fan Identification Scale**	**Total**	1 day^a^	132	37,74**±**14,03	1,383	,254	-
		2 days^b^	32	41,66**±**11,93			
		3 days and above^c^	14	41,29**±**9,02			

*p<,050

**p<,001

Upon examination of Table 8, it is evident that a significant positive correlation exists between the hate sub-dimension of the fan love-hate scale and the total score of the sports fan identification scale (r =, 611;p =, 000). Additionally, a moderate positive correlation is observed between the love dimension and the total score of the sports fan identification scale (r =, 591;p =, 000). Furthermore, a strong positive correlation is found between the total score of the fan love-hate level and the level of sports fan identification (r =, 776;p =, 000).

**Table 8:** Correlation between the fan love-hate and the fan identification.

			1	2	3	4
**Fan Love-Hate Scale**	**Hate**	**r**	**1**			
		**p**	**-**			
	**Love**	**r**	,202	1		
		**p**	,007	-		
	**Total**	**r**	,895	,548	1	
		**p**	,000	,000	-	
**Fan Identification Scale**	**Total**	**r**	,611	,591	,776	1
		**p**	,000	,000	,000	-

## 4. Discussion

The objective of this study is to examine the dynamics of the love-hate and identification relationships among those who engage in recreation purposes of basketball matches.

The study’s analysis of the demographic distribution of participants offers a understanding of the crowd composition at basketball matches. Upon conducting a comparative analysis with existing literature, similarities become evident. The research findings indicate that 68.0% of the individuals identified as male, whereas 32% identified as female. The prevalence of male domination aligns with previous research indicating that the viewership of basketball competitions primarily consists of men [31, 32]. This implies that basketball, when observed as a kind of entertainment, may possess a greater level of attractiveness among the male population. Our findings indicate a near-even split between single (53.4%) and married (46.6%) participants. While the literature does not provide a direct comparison for basketball spectators, it’s worth noting that marital status can influence recreational activities and choices. Moreover, it bears similar to the research conducted on leisure activities [33, 34]. The age distribution of the participants shows a higher concentration in the 26–35 age bracket, which is consistent with [31] result that many basketball match attendees are between 19–35 years old. Furthermore, our study has parallels with other studies in the existing literature [35, 36]. This age group might represent the most active and engaged demographic in terms of basketball viewership. A significant proportion of the participants, specifically 56.2%, has a university degree. Studies have discovered significant distinctions between education level and recreational activities [31]. This indicates that the level of education may influence the participation of spectators who watch sports for recreation.

The demographic data of the basketball spectators in our study provide information that is consistent with and extends previous global research. The finding that 68.0% of the participants were male and 32% were female is in line with [37] observation that gender has significant effects on basketball participation, reinforcing the sport’s greater appeal among men [38]. Emphasize the nuanced role of gender identity by adding that women’s identification with their gender affects their team identification and participation. The nearly equal distribution between single and married participants in your study brings a new perspective on the potential impact of marital status on sports spectatorship, an area that has not been extensively addressed in the existing literature. The age distribution data in your study, concentrated in the 26–35 age range, is consistent with previous findings that young adults (19–35 years) are the most active basketball spectators. In addition, the high proportion of respondents with a university degree (56.2%) supports the observed correlation between higher levels of education and increased recreational sports viewing. Overall, your study supports and enriches existing knowledge on how demographic factors such as gender, marital status, age and education level shape the spectator experience in sport.

A notable proportion of participants, equal to 74.2%, take part in the attendance of basketball matches on a weekly basis for a duration of one day. A smaller percentage of participants, comprising 18.0%, attend matches for a period of two days, while an even smaller proportion, accounting for 7.9%, attend matches for three or more days. This observed pattern may suggest that despite the widespread popularity of basketball, individuals’ frequent attendance may be constrained by various other commitments or personal interests. According to [31], it was also emphasized that a substantial number of spectators’ view basketball games as a recreational activity to be enjoyed in the company of loved ones, as opposed to merely a way to demonstrate support for their respective teams. Our findings are in line with research on soccer fans in Europe, where frequency of participation is often influenced by personal and occupational differences [36]. However, in contrast, studies of cricket fans in India show a higher frequency of participation, often linked to the social and festive nature of the sport [39]. This highlights a cultural difference where certain sports may have different priorities in people’s lives depending on the culture in the society.

Our findings are consistent with those of studies that have found significant gender differences in the subcategories of respect and hatred among university students [40]. This indicates that gender may influence the emotions and loyalty of sports fans toward their favorite teams. Our findings are also consistent with those of other studies [41, 42] that observed a significant difference between hatred and gender. The significant gender differences in emotional responses observed in our study are consistent with international research, such as studies on soccer fans in the UK, where male fans show higher levels of emotional involvement [27]. This pattern appears to be universal across different sports and cultures, suggesting inherent differences in how men and women engage with sports fandom.

Our study’s findings indicate that the marital status of fans does not significantly affect their feelings of love and hate towards the teams they support. This conclusion diverges from existing literature, which suggests a notable impact of marital status on fan identification and loyalty. For instance, a study conducted in the Eastern and South-eastern Anatolia Region found that married fans showed lower levels of identification with football clubs compared to their single counterparts [43]. Similarly, research in the United Kingdom revealed that single fans exhibited higher emotional engagement with sports teams than married fans [44].

Our findings indicate a significant difference in the hate sub-dimension of the Fan Love-Hate Scale between the age groups 26–33 and 30–35, as well as 26–33 and 36 and older. This indicates that the intensity of negative emotions or aversions toward opposing teams or players may vary by age group. This observation is consistent with the findings of [40], who discovered statistically significant differences between two sub-dimensions of respect and hatred in relation to several demographic variables, including age. Interestingly, no substantial difference was observed in the love dimension based on age. This indicates that positive feelings or allegiances toward a team or player remain consistent across age categories. The age-independent universality of affection or positive feelings toward a sports entity may be evidence of the unifying power of sports. The total score on the Sports Fan Identification Scale revealed significant age-based differences, particularly between the age groups 26–33 and 30–35, and between 26–33 and 36 and older. This finding is consistent with the work of [43], who analysed the level of identification of football fans with their teams in various regions and found age and other demographic differences. Also, the observed differences in the hate sub-dimension of the Fan Love-Hate Scale between age groups align with various research findings on the subject. For instance, [26] adapted the Love-Hate Scale for Sports Fans to Turkish, finding significant results regarding the validity and reliability of the scale for measuring love and hate among football fans across different age groups. Similarly, [6] examined the impact of aging on fan aggression, finding that older fans exhibited lower levels of fan hatred and aggression, supporting the idea that negative emotions towards opposing teams decrease with age.

No statistically significant differences were found in the sub-dimensions of the Fan Love-Hate Scale based on the education level of the participants, per our findings. This observation is consistent with [43] study. In addition, their research revealed no significant differences in fan identification based on educational level. Similarly, our study found no statistically significant difference in the total Sports Fan Identification Scale score based on educational level. Some studies emphasized that several factors influence follower identification, but education level may not be a major factor [45, 46].

Our findings indicate no statistically significant difference between the sub-dimensions of the Fan Love-Hate Scale and the frequency of attending sporting events. This suggests that the frequency with which fans attend sporting events does not affect the intensity of their love or hatred. This aligns with research from the University of Akron, which found no significant correlation between attendance frequency and fan identification [47]. However, other studies offer different perspectives. Research on sport spectatorship and life satisfaction indicates that individuals with higher levels of team identification perceive greater emotional support from other fans, suggesting a more complex relationship between live spectating and emotional outcomes [48]. Additionally, the psychology and motivations of sports fans suggest that while frequency of attendance may not directly influence fan emotions, underlying motivations and team identification can significantly impact emotional reactions [49].

The high positive correlation (r = .611, p .001) between the hate sub-dimension of the Fan Love-Hate Scale and the total score of the Sports Fan Identification Scale indicates that fans with greater levels of hatred toward rival teams or players are likely to have a stronger identification with their own team. This finding is consistent with [50] Social Identity Theory, which proposes that individuals derive their self-esteem from their group memberships. Antagonism toward rival groups can reinforce the boundaries between us (in-group) and them (out-group), bolstering identification with the in-group. Additionally, prior research [51] has demonstrated that rivalry intensity can increase group cohesion and identification among sports supporters.

The moderately positive correlation (r = .591, p .001) between the love dimension and the total score of the Sports Fan Identification Scale suggests that affectionate feelings toward one’s own team also contribute to the level of team identification, albeit to a lesser extent than the hate dimension. This is in line with past studies that have shown that positive emotions and attachment towards a team can cultivate a sense of belonging and identification among fans [27].

Moreover, the high positive correlation (r = .776, p .001) between the total score of the Fan Love-Hate level and the Sports Fan Identification level highlights the complex interaction between love and hate emotions in moulding the fan’s identification with their sports team. This indicates that both affiliative and antagonistic feelings toward teams or individuals in the sports domain are crucial determinants of fan identification. The dual role of love and hatred in fan identification recalls the Dualistic Model of Passion [52], which proposes that both positive and negative emotions can fuel a person’s passion and engagement in a specific domain. This is supported by the study from [53], which models discrete emotions as outcomes of team identification.

## 5. Conclusions

The aim of this study was to explore the dynamics of love-hate and identification relationships among spectators of basketball games for recreational purposes. Our findings reveal important demographic trends that contribute to a comprehensive understanding of the basketball spectator profile and provide insights into the broader dynamics of sports fandom.

Firstly, the demographic analysis highlights a predominance of male spectators (68%), which aligns with existing literature showing higher male engagement in basketball viewership. The nearly equal split between single (53.4%) and married (46.6%) participants, along with the concentration of spectators in the 26–35 age bracket, suggests that basketball appeals mainly to younger adults and shows the influence of marital status on recreational activities.

The educational background of participants, with 56.2% holding a university degree, indicates a correlation between higher education levels and sports spectatorship. This finding supports existing research linking education with recreational sports participation, suggesting that education may influence the motivations and patterns of sports engagement.

Our study also highlights the frequency of attendance at basketball matches, with 74.2% of participants attending weekly for one day. This pattern suggests that while basketball is popular, other commitments may limit how often spectators can attend. This finding is consistent with studies on soccer and cricket spectators, showing cultural variations in sports engagement.

The emotional dynamics of sports fandom were examined using the Fan Love-Hate Scale. Significant gender differences in emotional responses, particularly in respect and hatred, align with international research, suggesting inherent gender-based differences in sports fandom. Interestingly, marital status did not significantly affect fans’ feelings of love and hate towards their teams, diverging from some existing literature but highlighting the complexity of fan identification factors.

Age-based distinctions in the intensity of negative emotions towards rival teams and players were evident, with significant differences between younger and older age groups. This finding supports the idea that younger fans may exhibit higher levels of emotional engagement, which can be crucial for targeted audience engagement strategies.

The study found no significant differences in fan identification based on educational level or frequency of attendance, indicating that these factors may not significantly influence the intensity of emotional responses among fans. However, strong positive correlations were observed between both the love and hate dimensions of the Fan Love-Hate Scale and the total score of the Sports Fan Identification Scale. This emphasizes that both positive and negative emotions are integral to fan identification, supporting theories such as the Social Identity Theory and the Dualistic Model of Passion.

Overall, our study enhances the understanding of how demographic factors, emotional dynamics, and attendance patterns shape the spectator experience in basketball. The findings suggest that targeted strategies to enhance fan engagement should consider these demographic and emotional nuances.

Theoretically, these results contribute to a deeper understanding of fan identity. Practically, these insights offer valuable information for sports marketers and team management, highlighting the need for targeted strategies that cater to different demographic groups.

This study offers a foundational understanding of the affective dynamics among basketball fans. It opens several paths for future research that can expand the knowledge base and practical applications of sports fan psychology:

- Future research could explore a wider range of demographic factors, such as cultural background and socio-economic status, to understand their impact on fan emotions and identification.
- Since the study found more male basketball viewers, marketing should focus on activities and promotions that appeal to men.
- With many spectators aged 26–35, engagement strategies should target this active group. Social media campaigns, interactive digital experiences, and live event promotions could attract younger adults.
- Given that many spectators have university degrees, partnering with educational institutions for special events or offering discounted tickets for students and alumni could be beneficial.
- As 74.2% of participants attend weekly for one day, sports organizations should offer flexible ticket packages for different attendance frequencies.
- Since many spectators see basketball as a fun activity with loved ones, family and group discounts could encourage more frequent attendance.
- Considering significant gender differences in respect and hatred, fan engagement should address these emotional aspects. Tailored content that promotes respectful rivalry and positive interactions can help manage negative emotions. Fan zones and community-building events can encourage respectful support for teams.
- Strong correlations between fan love-hate emotions and identification suggest that community-building initiatives can strengthen fan loyalty. Creating spaces for fans to share their experiences, like online forums, social media groups, and fan conventions, can deepen their connection with the team.
- Improving the overall fan experience at basketball games with technological advancements like augmented reality, mobile apps, and interactive in-stadium experiences can attract and keep spectators. Ensuring these enhancements cater to diverse fan segments will maximize their impact.
